# Numerical Study of the Micro-Jet Formation in Double Flow Focusing Nozzle Geometry Using Different Water-Alcohol Solutions

**DOI:** 10.3390/ma14133614

**Published:** 2021-06-28

**Authors:** Grega Belšak, Saša Bajt, Božidar Šarler

**Affiliations:** 1 Laboratory for Fluid Dynamics and Thermodynamics, Faculty of Mechanical Engineering University of Ljubljana, 1000 Ljubljana, Slovenia; grega.belsak@fs.uni-lj.si; 2 Deutsches Elektronen-Synchrotron DESY, 22607 Hamburg, Germany; sasa.bajt@desy.de; 3 The Hamburg Centre for Ultrafast Imaging, 22761 Hamburg, Germany; 4 Laboratory for Simulation of Materials and Processes, Institute of Metals and Technology, 1000 Ljubljana, Slovenia

**Keywords:** micro-jets, double flow focusing nozzle, water + ethanol mixture, water + 2-propanol mixture

## Abstract

The purpose of this work is to determine, based on the computational model, whether a mixture of a binary liquid is capable of producing longer, thinner and faster gas-focused micro-jets, compared to the mono-constituent liquids of its components. Mixtures of water with two different alcohols, water + ethanol and water + 2-propanol, are considered. The numerical study of pre-mixed liquids is performed in the double flow focusing nozzle geometry used in sample delivery in serial femtosecond crystallography experiments. The study reveals that an optimal mixture for maximizing the jet length exists both in a water + ethanol and in a water + 2-propanol system. Additionally, the use of 2-propanol instead of ethanol results in a 34% jet length increase, while the jet diameters and velocities are similar for both mixtures. Pure ethanol and pure 2-propanol are the optimum liquids to achieve the smallest diameter and the fastest jets. However, the overall aim is to find a mixture with the longest, the smallest and the fastest jet. Based on our simulations, it appears that water + 2-propanol mixture might be slightly better than water + ethanol. This study reveals the dominant effect of liquid viscosity on the jet breakup process in a flow focusing nozzles operated under atmospheric conditions.

## 1. Introduction

Microfluidics explores the phenomena associated with liquid materials at microscopic scales where the typical sizes are measured in micrometres. These micro fluidical structures take many different forms, ranging from bubbles, droplets, jets, and sheets [1]. The related structures are found extensively in nature, but they can also be made in a controlled laboratory environment. Production of micro-jets is essential for serial femtosecond crystallography (SFX) experiments [2]. These experiments are typically performed with X-ray free electron lasers (XFELs), which enable collecting useful data on submicron crystals. In these experiments, a liquid micro-jet delivers organic nanocrystals into the pulsed X-ray beam. High intensity X-rays diffract of the protein crystals before their destruction and form diffraction images, which are recorded on a high-speed detector. From a large number of such images a precise structure of the organic nanocrystal can be determined. A stable jet, which delivers these crystals into the interaction region, is essential for a successful data collection and, over the years, many different delivery techniques have been developed. An in-depth overview of available injection techniques with assessment of their advantages and drawbacks is reported in [3]. Our paper focuses on pneumatically driven delivery systems.

The requirement of a thin, fast and long jet with a minimum sample consumption, suitable for SFX experiments, is currently practized with a double flow focusing nozzle (DFFN) [4]. The basic idea of the DFFN is described as follows. The primary central capillary used to deliver the liquid with nanocrystals is inserted into a capillary for secondary focusing liquid while both are inserted into the gas capillary. The secondary liquid has to be miscible with the primary fluid. This is to avoid unwanted jet instabilities due to immiscible fluids [4]. At the interface between the primary and the secondary liquid, the two fluids mix in such a way that the resulting mixed liquid produces thin and long jets with a minimum sample volumetric flow rate. Surface tension tends to break up the jet into droplets, thus it is beneficial to lower it to get longer jets. Viscosity, on the other hand, functions as a suppressor of the jet, breaking oscillations exerted by the high-speed gas on a liquid interface. Therefore, to obtain long jet high liquid viscosity is required. Hence, the main beneficial factors of the mixed primary and secondary liquid in DFFNs are their lower surface tension and higher viscosity. It should be noted that with the use of alcohols as secondary fluids, excessive mixing is undesirable due to the damage it may cause to the biological samples. A further benefit of DFFN is its ability to use low liquid flow rates for primary capillaries (stable jets are possible already at few μL/min flow rates), which reduces sample consumption of hard to prepare protein crystals. This is possible because the liquid emerging from the secondary capillary can produce a stable jet even at zero flow rate from the primary capillary [3]. Thus, the DFFN two-capillary configuration allows for a low primary liquid flow rate without losing the jet stability. Jet diameters obtained at such low liquid flow rates might be smaller than the protein crystal itself. DFFN nozzles are popular in SFX experiments because of the many benefits, including reduced sample consumption, reduced clogging of the nozzles, and easier jetting into a vacuum without ice formation, as discussed in more detail elsewhere [4].

On the numerical side, many attempts have been made to simulate the formation of the jet [5]. The droplet formation process in compound jets in axis symmetry was analyzed in [6], while the study in [7] performed detailed simulations of a microfluidic flow-focusing device. A gas dynamic virtual nozzle (GDVN) [8] model with a compressible gas flow was introduced to more accurately describe the jet flow and found a reasonable agreement with the experimentally obtained jet diameters [9]. Additionally, sensitivity studies of liquid properties [10], gas properties [11], nozzle structure [12] and nozzle outlet orifice design [13] were performed. Multiphase flows involving a single gas phase and two liquid phases have been previously numerically investigated in [14], where a jet breakup in a system of the compound jets in a gravitational field was simulated. In a flow focusing study with DFFN, analysis of the incompressible simulations involving a miscible and immiscible liquids was reported and showed that jets of miscible liquids are generally longer [4].

The main purpose of this paper is to understand the impact caused by the use of different fluids in a DFFN configuration on the jet diameter, velocity and length. The outline of the paper is the following: Section 2 introduces the physical model and explains the numerical solution. The results are given in Section 3 and the discussion is presented in Section 4.

## 2. Materials and Methods

### 2.1. Pre-Mixed Double Flow Focusing Nozzle

**Figure 1:**
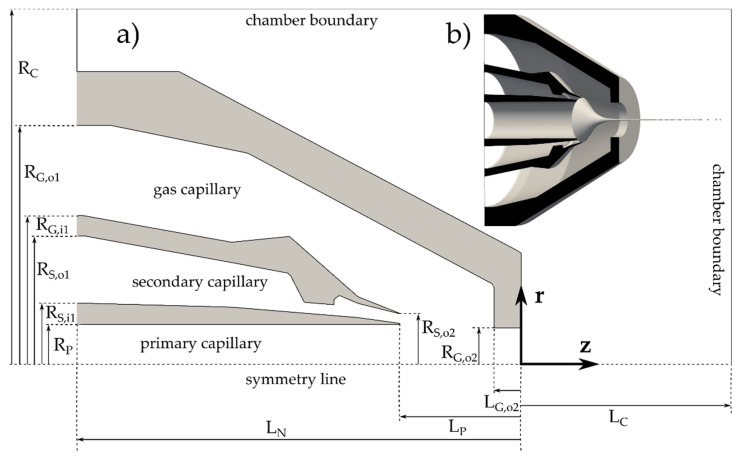
(**a**) A 2D section of a DFFN with relevant dimensions; (**b**) rotated view of a DFFN sliced in half, with a depicted jet leaving the nozzle and breaking it up. The nozzle design is taken from [15].

The DFFN design, located in a pressure-controlled chamber, consists of three coaxial capillaries (Figure 1). The outermost capillary is used for gas delivery. Its converging shape and a small outlet orifice allow for the compression and acceleration of the gas. The primary capillary delivers the sample. Another insertion places the secondary capillary coaxially around the primary capillary. The two liquids exiting their capillaries are heavily compressed and accelerated by the gas, exiting from the gas capillary, positioned coaxially around the secondary capillary. Such a DFFN setting produces a jet (about 10 μM in diameter and 100 μM in length) that exits the nozzle and breaks up downstream.

Figure 1b shows a slightly rotated side view of a nozzle structure, sliced in half. Axial symmetry allows for a description with the coordinates r and z, which are, along with relevant dimensions, plotted in part (a) of the figure. The dimensions in the radial direction, r, are denoted with R and an appropriate subscript. The subscripts denoting primary, secondary, gas, inner, outer, and chamber are marked as P, S, G, i, o, and C, respectively. Subscripts 1 and 2 relate to the extreme positions at the capillary, where the value 1 denotes the beginning of the certain capillary and 2 denotes the end. Thus, for example R_G,i1_ is the inner radius of the gas capillary at its inlet.

The radial coordinates of the nozzle structure are R_P_ = 36.5 μM, R_S,i1_ = 52.5 μM, R_S,o1_ = 105.5 μM, R_G,i1_ = 117.5 μM, R_G,o1_ = 184.5 μM, R_S,o2_ = 44.5 μM, R_G,o2_ = 34 μM and R_C_ = 10,000 μM, while the axial coordinates are L_P_ = −90 μM, L_N_ = −330 μM, L_G,o2_ = −20 μM and L_C_ = 19,670 μM.

The DFFNs are 3D printed [15,16], which ensures precise placement and alignment of all three capillaries. The unusual structure of the wall between the secondary and the gas capillary is related to the nozzle manufacturing constraints and stability. However, it does not affect the flow of either fluid in any substantial way. The related convex section of the wall can be omitted, and this has no effect on the numerical fluid–flow results. This is the reason why the dimensions of this section are absent, and only the relevant points are shown.

A primary volumetric liquid flow rate (Φ_P_) is imposed at the inlet of the primary capillary, while a secondary volumetric liquid flow rate (Φ_S_) flows through the secondary capillary. At the entrance of the gas capillary the gas mass flow rate m_g_ is set. All the simulations that follow are performed with the same mass flow rate of m_g_ = 17 mg/min and the same volumetric flow rates, Φ_P_ = 4.5 μL/min and Φ_S_ = 5.5 μL/min. The nozzle outlet plenum pressure is set to an atmospheric pressure of 10^5^ Pa.

The following set of independent dimensionless numbers fully describes the fluid flow: gas Reynolds number Re_g_ = ρ_g_U_avg,g_R_G,02_/µ_g_, liquid Reynolds number Re_l_ = ρ_l_U_avg,l_D_0_/µL, Weber number We = ρ_l_U_avg,l_^2^ R_G,02_/σ, capillary number Ca = µU_avg,l_/σ, ratio of liquid to gas mass flow rate Q = ρ_l_Φ_T_/m_g_, viscosity ratio µ_R_ = µL/µ_g_ and density ratio ρ_R_= ρ_l_/ρ_g_. Velocity U_avg_ is an average value of the velocity field either of the gas or the liquid, calculated over the cross-section at the nozzle exit (z = 0 μM). The properties of helium at a standard pressure and temperature are used in the calculation of dimensionless numbers. The explored dimensionless range of the system is presented in Table 1.

### 2.2. Computational Model

Typically, a DFFN uses two separate liquids. The first one is delivered through the primary capillary and the second one through the secondary one that surrounds the primary one. The liquids then meet at the meniscus, where they are mixed in the recirculation zone through the process of diffusion and forced mixing. Both processes continue downstream through the entire length of the jet. This results in a situation where the combined liquid has different physical properties at each position of the jet length, depending on the local amount of mixing. This point is important due to the fact that the breakup point of the jet strongly depends on its physical properties. The properties are changing until a fully mixed primary and secondary liquid is reached. 

In our model, we consider a mixture of liquids instead of two distinct liquids. This means that the two liquids are already mixed before entering the DFFN orifice. The same, a fully pre-mixed primary and secondary liquid is considered to be fed to the primary and the secondary capillary, simultaneously. This approach enables us to use a simple numerical model, allowing for the use of a compressible two-phase solver with only one surface between the gas and the liquid. This offers greater stability, accuracy and computing efficiency compared to the model where the mixing of both fluids would be explicitly considered. The main purpose of this study is to determine how different pre-mixed fluids behave in the DFFN geometry.

#### 2.2.1. Governing Equations

We assume a laminar Newtonian two-phase flow with a compressible gas and an incompressible liquid. Phase change (evaporation or freezing) of the liquid is not assumed. The flow is described by the mixture formulation of Navier–Stokes equations. The finite volume method [17] is employed along with the volume of fluid (VOF) method [18]. The VOF method solves for a numerical volume fraction field (*α*), which is defined as a ratio of the volumetric amount of one fluid in a cell with the total volume of that cell. This defines the interface between the fluids, which is advected in the velocity field (***u***) by the interface advection equation:

(1) $$\frac{\partial \alpha }{\partial t}+\nabla \cdot (\alpha u)=0$$

Geometric VOF [19] is used to solve Equation (1). The mixture formulation defines the material properties (Ψ) as $\Psi =\alpha \Psi_{l}+(1-\alpha )\Psi_{g}$, where subscript g denotes the material property of the gas and l of the liquid. The material properties are density, *ρ*, dynamic viscosity, *μ*, specific heat, $c_{p}$ and thermal conductivity, k. The calculated equations of mass, momentum and energy conservation for Newtonian fluids with the addition of ideal gas equation are the following:

(2) $$\frac{\partial \rho }{\partial t}+\nabla \cdot (\rho u)=0$$

(3) $$\frac{\partial (\rho u)}{\partial t}+\nabla \cdot (\rho uu)=-\nabla p+\nabla \cdot \left[\mu \left(\nabla u+\nabla u^{T}\right)-\frac{2}{3}\mu \left(\nabla \cdot u\right)I\right]+\sigma \kappa \nabla \alpha$$

(4) $$\frac{\partial (\rho c_{V}T)}{\partial t}+\nabla \cdot (\rho c_{V}Tu)+\frac{\partial (\rho \frac{1}{2}{\left|u\right|}^{2})}{\partial t}+\nabla \cdot (\rho \frac{1}{2}{\left|u\right|}^{2}u)+\nabla \cdot (up)=\nabla \cdot (k\nabla T)$$

(5) $$\rho_{g}=\frac{pM}{RT}$$

Pressure, identity tensor, surface tension, curvature, temperature, molar mass, and universal gas constant are denoted by p,I,σ,κ,T,M and R, respectively. The curvature is calculated through the divergence of the scaled volume fraction gradient as:

(6) $$\kappa =-\nabla \cdot (\nabla \alpha /\left|\nabla \alpha \right|)$$

For the purpose of solving this model, we utilize the solver compressibleInterIsoFoam provided by OpenFOAM software, Ver. 8, released 22 July 2020 [20].

#### 2.2.2. Computational Domain and Boundary Conditions

The mixture study presented in this paper evaluates three different characteristic jet parameters: diameter, velocity, and length. The spatial discretization of the computational domain is set up in such a manner that the gas and the liquid flows are sufficiently resolved inside and outside of the nozzle structure. Finer refinement is chosen near the symmetry line where the liquid flows and gas-liquid interface is expected. The diameter, D_0_, is measured at the nozzle orifice exit, which is defined as z = 0. The length of the jet, L, is represented as a distance of the z coordinate from the nozzle exit to the end of the jet at r = 0. Velocity U_100_ is a z component of the velocity at the center point of the jet at the position z = 100 μM. All three parameters are temporarily averaged values over a 50-µs real time, at a sampling interval of 0.125 µs. This represents a dataset of 400 points. To ensure that the chosen time interval is large enough and, thus, an acceptable representation of the results, one simulation is run for three different time intervals: 25 µs, 50 µs and 75 µs. The average values of diameter, velocity and length do not change substantially, with the increase of the data points from 400 to 600. The diameter increases by 0.1%, velocity decreases by 0.2% and length increases by 0.4%. Thus, an interval of 50 µs is deemed as satisfactory and is used in all other simulations. 

**Figure 2:**
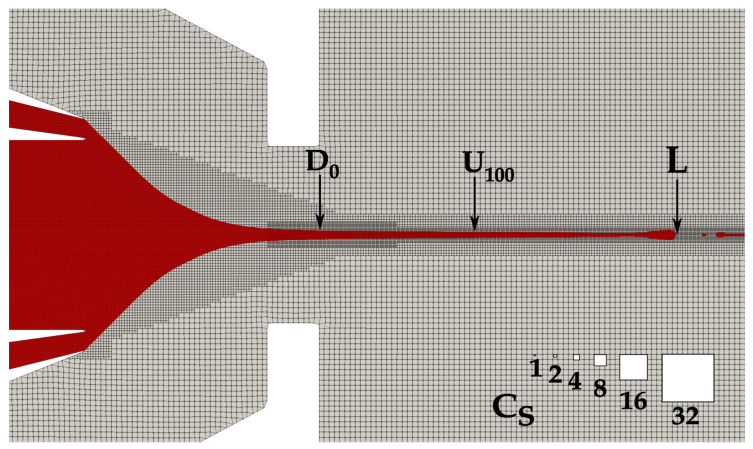
Representation of the utilized mesh along with the three characteristic jets parameters: diameter, velocity and length. White boxes show the normalized cell sizes. The image is zoomed- in to show only the most interesting details.

To simplify comparison of the diameter, length, and velocity of jets created by mixtures and pure water the results are normalized throughout the text with values corresponding to the pure water. The normalization values for length, diameter and velocity of pure water jets are L_N_ = 234.1 µm, D_N_ = 3.3 µm and U_N_ = 39.9 m/s, respectively. A cell size normalization value of C_S,N_ = 0.25 µm is taken, which also represents the smallest cell size used in this study. The cell size between the two sequential refinement layers differs by a factor of 2. Normalized cell sizes in the mesh have an interval of C_S_ = 1256. The largest cell sizes are located far from the nozzle and cannot be seen in Figure 2. Figure 2 is a zoomed-in view, focused on the nozzle details.

This arrangement and density of the mesh assure reasonable mesh independent results. A related study is given in [9]. The axial symmetry of the nozzle allows for the simulations to be run on a two-dimensional wedge type static mesh, whose wedge angle is set to 4°, as recommended in the OpenFOAM user’s guide [20]. Velocities at the inlets of primary and secondary capillaries are prescribed with the volumetric flow rates Φ_P_ and Φ_S_, while the velocity at the inlet of the gas capillary is specified through a mass flow rate m_g_. These three conditions impose a constant inlet profile. To avoid errors and to obtain the developed velocity profiles, sufficiently long inlet capillaries are chosen. Velocity at the walls is set to no-penetration and no-slip condition, and at the outlet, *pressureInletOutletVelocity* boundary condition is used. This mixed condition sets a zero gradient value for the outflow and a fixed value for the inflow. A fixed temperature of 293 K is imposed on the walls and inlets, while *inletOutlet* condition is set at the outlets. This mixed condition imposes a zero-gradient condition for outflow and a user-specified fixed value for reverse flow, which is set to zero. The pressure boundary condition set at the outlet chamber boundaries is a constant *totalPressure*, which imposes a fixed pressure value, the value of which is set to a pressure of 1 bar. Walls and inlets are prescribed *fixedFluxPressure* (fixed pressure gradient) condition. Volume fraction field alpha is set to *zeroGradient* at the walls and *inletOutlet* with an initial value of 1 at the liquid capillary inlets and a value of 0 elsewhere. The boundary conditions, denoted in italics, correspond to the standard definitions of the boundary conditions in OpenFOAM.

A mesh independence study on the formation of micro-jets under comparable operating conditions and based on the same numerical model [9], determined the necessary resolution for a mesh independent results of diameter and length. There it was concluded, through the use of Richardson extrapolation, that normalized cell size of Cs = 2 is sufficient for diameter convergence. In contrast, a normalized cell size of Cs = 1 is needed to achieve converging lengths. Based on that study we also utilize the resolution Cs = 1 to ensure both diameter and length mesh independence. 

#### 2.2.3. Solution Procedure and Computer Hardware

The continuum surface force model [21], which explicitly implements the surface tension force in the momentum equation (3), is used. The temporal resolution follows the CFL condition [22], where the Courant number is set to 0.95. The first order implicit Euler temporal scheme is chosen. The Gauss linear scheme is used for the gradient terms, while the Laplace operators are discretized with a Gauss linear corrected scheme. The convective terms in the momentum and energy equation are discretized with a first-order bounded upwind scheme. The diffusivity terms are set to the second-order unbounded linear scheme. The reader is referred to the OpenFOAM user guide [20] for additional details on the specific discretization scheme used in the present work. The system of equations is solved by preconditioned conjugate gradient, geometric–algebraic multigrid and diagonal incomplete Cholesky solvers [23]. The pressure–velocity coupling is operated in the PISO mode [24]. 

In total, 13 simulations were performed. All simulations were run in parallel on an HPC computer at the Faculty of Mechanical Engineering of the University of Ljubljana using 24 processors (2 × 12-cores Intel Xeon E5-2680V3 processor with a working speed of 2.5 GHz). Together, this accounts for roughly 2 months of computational time.

#### 2.2.4. Liquid Properties

The liquids considered here are different mixtures of water + ethanol and water + 2-propanol. 2-propanol is known in the scientific literature by many names, such as isopropanol, rubbing alcohol or dimethyl carbinol, to name a few. The simulation relevant liquid properties for a specific mixture are mole fraction X_i_, surface tension, density, dynamic viscosity, molar weight, specific heat and Prandtl number. A liquid with X_i_ = 0 represents pure water, while a liquid with X_i_ = 1 represents pure ethanol in water + ethanol mixture or pure 2-propanol in water + 2-propanol mixture, respectively. The set of water + ethanol mixture data is presented in Table 2. Surface tension, density, dynamic viscosity and molar weight for each of the nine sets of mixture data defined in this way are based on measured values [25]. The thermal conductivity values are also experimentally determined [26] and are used in the calculation of the Prandtl number. The values of specific heat were calculated through the mixture rule.

The liquid properties of six mixtures in water + 2-propanol are shown in Table 3. Experimentally determined surface tension values are taken from [27], while density, dynamic viscosity and molar weight are taken from [28]. Specific heat values are taken from [29]. 

The data from Table 2 and Table 3 show that with increasing ethanol or 2-propanol content, the density is gradually decreasing while the molar weight is increasing. Specific heat decreases steadily in water + ethanol system while in water + 2-propanol system it shows a slight increase at a low molar fraction of 2-propanol and falls afterwards. Surface tension falls quickly with a small addition of either ethanol or 2-propanol and nearly plateaus towards the pure alcohol. The dynamic viscosity exhibits unique behaviour. A small addition of alcohol at first increases its value, but then the dynamic viscosity gradually gets smaller approaching the value in pure alcohol. The peak viscosity is achieved at X_i_ = 0.312 in water + ethanol system and at X_i_ = 0.259 in water + 2-propanol system. The two alcohols reach this peak at different molar fractions. The peak viscosity value of water + 2-propanol is roughly 25% higher than in water + ethanol. Selected molar fractions values in Table 2 and Table 3 differ slightly, because they were chosen in a manner to best to describe the viscosity peak, which lies at different molar fractions in the two mixtures.

## 3. Results

### Water + Ethanol and Water + 2-Propanol Mixtures

**Figure 3:**
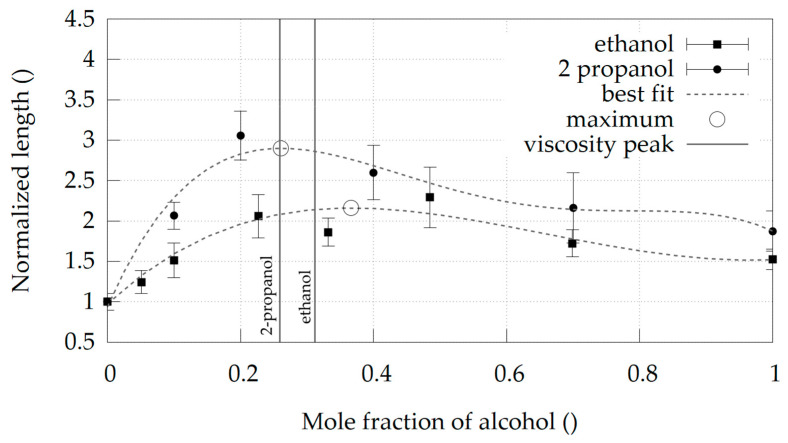
Normalized jet length at various molar fractions X_i_ for two different alcohols. Black squares and dots denote the average length values, while the two dotted lines depict corresponding best fits to the available dataset (fourth-order polynomial). The maximum length in each system is marked by an empty circle. Black vertical lines denote the molar fraction at which a corresponding mixture reaches a viscosity peak. Error bars represent standard deviation.

The average jet length, L, in mixtures with different molar fractions for two different alcohols is shown in Figure 3. The observed length follows a similar trend as the dynamic viscosity. The increase of alcohol content initially prolongs the jet until the maximum length is reached, while a further increase of alcohol content shortens the jet. This is true for both systems, the water + ethanol and the water + 2-propanol. The peak length, determined as a maximum of a fourth (4th) order polynomial fit to the data, is achieved at X_i_ = 0.366 in the water-ethanol system and at X_i_ = 0.261 in the water + 2-propanol system. 

The molar fraction values of the two jet length peaks coincide with the molar fraction values of the viscosity peaks. The molar fraction difference between the peak length and the peak viscosity (X_i,peak length_ − X_i,peak viscoisty_) is 0.054 for water + ethanol system and 0.002 for water + 2-propanol system. Therefore, we conclude that the jet length primarily depends on the viscosity of the liquid. Figure 3 also shows that a mixture of water + 2-propanol can produce longer jets than a mixture of water + ethanol. The maximum jet length of water + 2-propanol mixture is 34% longer than the maximum length of water + ethanol and this maximum can be reached at lower alcohol contents. The optimum water + ethanol mixture can produce twice as long jets, while in water + 2-propanol system, the jets can be nearly three times longer than with pure water.

**Figure 4:**
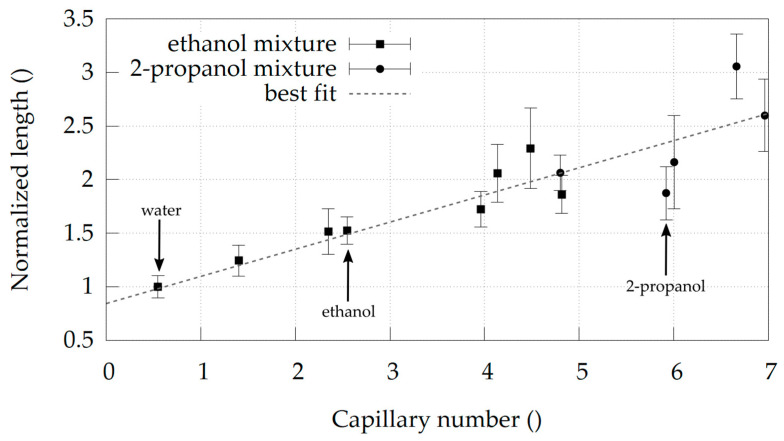
Normalized jet length as a function of capillary number. Black squares denote different water + ethanol mixtures, while black circles correspond to different water + 2-propanol mixtures. Pure liquids are marked with an arrow. The dotted line depicts the best linear fit to the available dataset.

Additionally, the strong dependence of the micro-jet breakup process on the viscous forces is demonstrated in Figure 4, where different mixtures of ethanol and 2-propanol and their corresponding lengths are plotted against the dimensionless capillary number. From all the various mixtures examined, pure water is the only one that has a capillary number smaller than one. There, the surface tension forces are dominant. In all other fluids, the viscosity is the leading property dictating the jet breakup. Figure 4 demonstrates the increase in jet length with the rise in jets capillary number.

**Figure 5:**
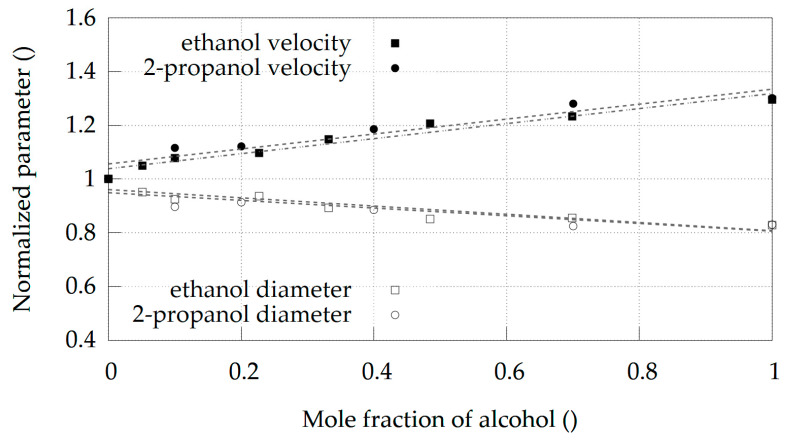
Normalized jet diameter, D_0_, and velocity, U_100_, at various molar fractions, X_i_, for two different alcohols. Filled circles and squares denote the velocities, while empty ones show the diameters. The dotted lines depict the corresponding best fit to the available dataset (linear function).

The diameters (D_0_) and velocities (U_100_) for both alcohols and their respective molar fractions are shown in Figure 5. With the increasing alcohol content, the diameter of the jets decreases. A linear fit shows that a pure ethanol jet provides a 17% thinner jet while pure 2-propanol exhibits a 16.5% thinner jet compared to pure water. With the increasing molar fraction, the jet velocities also increase. Pure ethanol and pure 2-propanol jets are around 30% faster than pure water jets. 

## 4. Discussion

One of the main conclusions of the present work is that the length of micro-jets in the DFFNs operated under the atmospheric conditions is predominantly determined by the liquid viscosity, with higher viscosity values being associated with longer jets. This gives insights into why DFFNs produce longer jets than a GDVN nozzle. GDVNs only use a single liquid, typically water, as a delivery fluid and, therefore, cannot benefit from mixing with the secondary liquid that can lead to increased viscosity. We showed that adding a small amount of alcohol (ethanol or 2-propanol) to water has a beneficial effect on prolonging the jet and that there is an optimum mixture where this effect is most pronounced. If the DFFN is operated under the conditions that the emerging jet reaches an optimum mixture of the primary and secondary fluid, the maximum jet length will be produced. Thus, the optimization procedure for achieving the maximum jet length with DFFN is to find the exact molar fraction value for a mixture with the highest viscosity value and enforcing it through the setup of operating conditions. 

As a secondary liquid, we explored ethanol and 2-propanol. Our simulations indicate longer jets for water + 2-propanol as compared to water + ethanol mixture. This also happens at a lower 2-propanol molar fraction. Since alcohol can be damaging to the organic crystals investigated in SFX experiments, a mixture with lower alcohol content is of greater practical value. The water + ethanol system achieves a maximum jet length at ethanol molar concentrations of X_i_ = 0.366, but according to Figure 4 the water + 2-propanol mixture achieves the same length at X_i_ = 0.085. This is roughly four times lower alcohol molar concentration than in the water + ethanol mixture. However, as explained at the start, jet length is not the only parameter of interest. For SFX experiments, it is also important that the jet diameter be as small as possible to reduce unwanted background from the delivery liquid and that the jet is very fast to replenish crystals in the interaction zone with fresh, unexposed crystals. Based on our simulations and checking the values in Figure 5, there is no significant difference in jet diameter and jet velocity compared to water + ethanol and water + 2-propanol at Xi = 0.085. The jet using water + 2-propanol mixture is slightly faster and has a smaller diameter compared to the jet using water + ethanol. This suggests that the use of water + 2-propanol mixture might be overall beneficial for protein crystals, assuming similar toxicity of ethanol and 2-propanol. In general, such simulations can greatly simplify the search for alternative secondary fluids in DFFNs when the length, velocity, diameter or other parameters of the jet are to be optimized. 

## Figures and Tables

**Table 1:** The dimensionless number range used in the simulations.

Quantity	Re_g_	Re_l_	We	Q	µ_R_	ρ_R_
Minimum	125.96	12.17	102.21	0.46	52.63	4741.4
Maximum	127.2	79.67	843.50	0.59	157.63	6009.6

**Table 2:** Physical properties of the water + ethanol mixture with corresponding ethanol molar fraction values at the temperature of 293 K. The datasets are taken from [24,25].

X_i_	Surface Tension (N/m) ×10^−3^	Density (kg/m^3^)	Dynamic Viscosity (kg/ms) ×10^−3^	Molar Weight (kg/mol)	Specific Heat (W/mK)	Prandtl Number
0	72.800	1000.000	1.000	18.000	4184	6.080
0.051	47.961	981.445	1.634	19.412	3966	13.083
0.100	39.443	967.026	2.181	20.792	3748	19.821
0.154	33.521	951.516	2.593	22.311	3530	26.447
0.227	31.743	931.372	2.903	24.366	3312	32.938
0.332	28.896	904.432	2.987	27.340	3094	37.440
0.485	26.410	871.692	2.671	31.606	2876	36.705
0.699	24.862	837.380	1.997	37.603	2658	29.722
1.000	22.300	789.000	1.120	46.000	2440	19.860

**Table 3:** Physical properties of the water + 2-propanol mixture with corresponding ethanol molar fraction values at the temperature of 293 K. The datasets are taken from [26,27,28].

X_i_	Surface Tension (N/m) ×10^−3^	Density (kg/m^3^)	Dynamic Viscosity (kg/ms) ×10^−3^	Molar Weight (kg/mol)	Specific Heat (W/mK)	Prandtl Number
0	72.800	1000.000	1.000	18.000	4184	6.080
0.100	27.369	956.672	2.947	22.223	4433	18.680
0.200	24.776	922.029	3.692	26.431	3941	24.226
0.400	23.869	864.935	3.514	34.847	3426	24.622
0.700	22.507	809.778	2.643	47.471	2989	19.778
1.000	21.146	791.200	2.411	60.095	2552	18.468

## Data Availability

The data presented in this study are available on request from the corresponding author.

## References

[1] Chakraborty S. (2010). Microfluidics and Microfabrication.

[2] Chapman H.N., Fromme P., Barty A., White T.A., Kirian R.A., Aquila A., Hunter M.S., Schulz J., DePonte D.P., Weierstall U. (2011). Femtosecond X-ray protein nanocrystallography. Nature.

[3] Boutet S., Fromme P., Mark S.H. (2018). X-ray Free Electron Lasers.

[4] Oberthuer D., Knoska J., Wiedorn M.O., Beyerlein K.R., Bushnell D.A., Kovaleva E.G., Heymann M., Gumprecht L., Kirian R.A., Barty A. (2017). Double-flow focused liquid injector for efficient serial femtosecond crystallography. Sci. Rep..

[5] Herrada M.A., Ganan-Calvo A.M., Ojeda-Monge A., Bluth B., Riesco-Chueca P. (2008). Liquid flow focused by a gas: Jetting, dripping, and recirculation. Phys. Rev. E.

[6] Liu X., Wu L., Zhao Y., Chen Y. (2017). Study of compound drop formation in axisymmetric microfluidic devices with different geometries. Colloids Surf. A Physicochem. Eng. Asp..

[7] Dupin M.M., Halliday I., Care C.M. (2006). Simulation of a microfluidic flow-focusing device. Phys. Rev. E.

[8] Ganan-Calvo A.M. (1998). Generation of Steady Liquid Microthreads and Micron-Sized Monodisperse Sprays in Gas Streams. Phys. Rev. Lett..

[9] Zahoor R., Belšak G., Bajt S., Šarler B. (2018). Simulation of liquid micro-jet in free expanding high-speed co-flowing gas streams. Microfluid. Nanofluid..

[10] Zahoor R., Regvar R., Bajt S., Šarler B. (2019). A numerical study on the influence of liquid properties on gas-focused micro-jets. Prog. Comput. Fluid Dyn. Int. J..

[11] Zahoor R., Bajt S., Šarler B. (2018). Numerical investigation on influence of focusing gas type on liquid micro-jet characteristics. Int. J. Hydromechatron..

[12] Zahoor R., Bajt S., Šarler B. (2018). Influence of Gas Dynamic Virtual Nozzle Geometry on Micro-Jet Characteristics. Int. J. Multiph. Flow.

[13] Šarler B., Zahoor R., Bajt S. (2021). Alternative Geometric Arrangements of the Nozzle Outlet Orifice for Liquid Micro-Jet Focusing in Gas Dynamic Virtual Nozzles. Materials.

[14] Van Vu T., Homma S., Wells J.C., Takakura H., Tryggvason G. (2011). Numerical Simulation of Formation and Breakup of a Three-Fluid Compound Jet. J. Fluid Sci. Technol..

[15] Knoška J., Adriano L., Awel S., Beyerlein K.R., Yefanov O., Oberthuer D., Murillo G.E.P., Roth N., Sarrou I., Villanueva-Perez P. (2020). Ultracompact 3D microfluidics for time-resolved structural biology. Nat. Commun..

[16] Nelson G., Kirian R.A., Weierstall U., Zatsepin N.A., Faragó T., Baumbach T., Wilde F., Niesler F.B.P., Zimmer B., Ishigami I. (2016). Three-dimensional-printed gas dynamic virtual nozzles for x-ray laser sample delivery. Opt. Express.

[17] Hsu C.-J. (1981). Numerical Heat Transfer and Fluid Flow. Nucl. Sci. Eng..

[18] Hirt C.W., Nichols B.D. (1981). Volume of fluid (VOF) method for the dynamics of free boundaries. J. Comput. Phys..

[19] Roenby J., Bredmose H., Jasak H. (2016). A computational method for sharp interface advection. R. Soc. Open Sci..

[20] Weller H.G., Tabor G., Jasak H., Fureby C. (1998). A tensorial approach to computational continuum mechanics using object-oriented techniques. Comput. Phys..

[21] Brackbill J.U., Kothe D.B., Zemach C. (1992). A continuum method for modeling surface tension. J. Comput. Phys..

[22] Courant R., Friedrichs K., Lewy H. (1967). On the Partial Difference Equations of Mathematical Physics. IBM J. Res. Dev..

[23] Datta B.N. (2010). Numerical Linear Algebra and Applications.

[24] Issa R.I. (1986). Solution of the implicitly discretized fluid flow equations by operator-splitting. J. Comput. Phys..

[25] Khattab I.S., Bandarkar F., Fakhree M.A.A., Jouyban A. (2012). Density, viscosity, and surface tension of water+ ethanol mixtures from 293 to 323K. Korean J. Chem. Eng..

[26] Matvienko A., Mandelis A. (2005). Ultrahigh-resolution pyroelectric thermal-wave technique for the measurement of thermal diffusivity of low-concentration water-alcohol mixtures. Rev. Sci. Instrum..

[27] Vazquez G., Alvarez E., Navaza J.M. (1995). Surface Tension of Alcohol+ Water from 20 to 50 °C. J. Chem. Eng. Data.

[28] Pang F.M., Seng C.E., Teng T.T., Ibrahim M.H. (2007). Densities and viscosities of aqueous solutions of 1-propanol and 2-propanol at temperatures from 293.15 K to 333.15 K. J. Mol. Liq..

[29] Arnaud R., Avedikian L., Morel J.P. (1972). Chaleurs molaires de quelques mélanges hydroorganiques riches en eau. J. Chim. Phys..

